# Gastrointestinal bleed mortality disparities in patients with atrial fibrillation: A cross‐sectional analysis 1999–2020

**DOI:** 10.1002/joa3.13223

**Published:** 2025-01-14

**Authors:** Enkhtsogt Sainbayar, Ramzi Ibrahim, Sangkyu Noh, Hoang Nhat Pham, Mahek Shahid, Joseph Elias, Harneet Grewal, Rama Mouhaffel, Akira Folk, Jack Hartnett, Kwan Lee, Justin Z. Lee

**Affiliations:** ^1^ Department of Medicine University of Arizona Tucson Tucson Arizona USA; ^2^ Department of Cardiovascular Medicine Mayo Clinic Phoenix Arizona USA; ^3^ Department of Cardiology, DeBakey Heart and Vascular Center Houston Methodist Houston Texas USA; ^4^ Department of Medicine Abrazo Health Network Glendale Arizona USA; ^5^ Department of Cardiovascular Medicine Cleveland Clinic Cleveland Ohio USA

**Keywords:** arrhythmia, atrial fibrillation, gastrointestinal, hemorrhage

## Abstract

**Introduction:**

Gastrointestinal bleeding (GIB) is often encountered among patients with atrial fibrillation (AF) due to the use of anticoagulation. This study assesses disparities in GIB‐related mortality among decedents with AF in the United States.

**Methods:**

GIB mortality data in patients with AF from 1999 to 2020 was queried from the CDC database. Decedent demographic information (age, sex, race and ethnicity, and geographic residence) was obtained from death certificates. We calculated age‐adjusted mortality rates (AAMRs) through the direct method and estimated the annual percentage change (APC) in mortality using log‐linear regression models.

**Results:**

From 11,209 GIB‐related deaths among AF decedents, we observed an increase in AAMR from 0.12 in 1999 to 0.21 in 2020, particularly during the 2009 to 2020 period (APC +4.8, *p* < .001). Disproportionate mortality rates were noted in males (AAMR 0.18) and White populations (AAMR 0.15) as compared to females (AAMR 0.13) and Black populations (AAMR 0.10), respectively. Rural regions also reported higher mortality (AAMR 0.18) than urban areas (AAMR 0.14). Mortality shifts in urban regions remained stagnant from 1999 to 2009 (APC –0.15, *p* = .806) followed by an increase from 2009 to 2020 (APC +4.83, *p* < .001). However, mortality increased consistently from 1999 to 2020 in rural regions (APC +4.08, *p* < .001). The Northeast US exhibited the highest mortality rate (AAMR 0.18), followed by the Midwest (AAMR 0.16), West (AAMR 0.14), and South (AAMR 0.13).

**Conclusions:**

Disparities in GIB mortality among AF decedents were identified. These findings accentuate the need for targeted interventions to mitigate GIB risks in vulnerable subgroups.

## Graphical abstract

Our results identified disparities in gastrointestinal bleed mortality among decedents with atrial fibrillation, emphasizing the importance of public health measures to mitigate healthcare inequities.

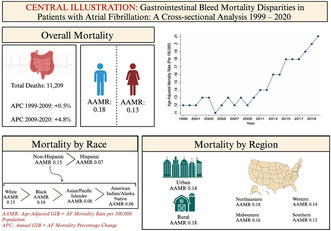

## 1 INTRODUCTION

Atrial fibrillation (AF) is the most common arrhythmia, affecting over 37 million individuals globally.^1^ The prevalence of gastrointestinal bleeding (GIB) presents a significant challenge in AF care, contributing to both morbidity and mortality. This is particularly concerning for patients on oral anticoagulants. Several factors have been associated with increased GIB risk in patients using warfarin and direct oral anticoagulants (DOACs), such as advanced age, simultaneous aspirin or NSAID use, and chronic kidney disease comorbidities.^2^, ^3^, ^4^, ^5^, ^6^ Other predictors of GIB mortality among patients with AF include overweight or obesity, prior history of GIB, baseline anemia, concomitant heart failure or malignancy, and polypharmacy.^4^, ^5^, ^6^ Despite this, the disparities in GIB‐related mortality among the AF patient population in the United States (US), particularly across different demographics and geographical regions, remain underexplored. Our study aimed to explore trends in GIB mortality among AF decedents, with an emphasis on exploring disparities linked to patient demographics and geographic locations within the US.

## 2 METHODS

We sourced death certificate data from the Centers for Disease Control and Prevention (CDC).

Wide‐ranging Online Data for Epidemiologic Research (WONDER) database.^7^ This data includes the primary cause of death, as defined by the World Health Organization—the diagnosis directly leading to or initiating the fatal sequence of events. It also includes contributing conditions that influenced the decedent's cause of death. Our search focused on deaths where GIB (Table S1) was listed as the primary cause of death and AF (International Classification of Diseases, Tenth Revision: I48) was a contributing factor from 1999 to 2020. The demographic information accompanying each record included the decedent's age, race and ethnicity, sex, and residence. We classified ethnicity into non‐Hispanic and Hispanic categories. Racial categories encompassed non‐Hispanic White, non‐Hispanic Black, non‐Hispanic American Indian/Alaska Native, and non‐Hispanic Asian/Pacific Islander adults. Residential areas were categorized by the 2013 National Center for Health Statistics urbanization scheme into urban or rural areas, along with the respective US census region (Midwest, Northeast, West, and South).

For each year, we computed crude annual mortality rates by dividing GIB and AF‐related absolute number of deaths by the population size. Age‐adjusted mortality rates (AAMR) were calculated using the direct method, standardizing to the 2000 US population. This was applied both cumulatively and across different demographic groups. To analyze temporal trends in the AAMR from 1999 to 2020, we used log‐linear regression models, permitting up to four inflection points for the 22‐year period (National Cancer Institute).^8^ The annual percentage change (APC) in mortality rates was determined using the Monte‐Carlo permutation test.^9^, ^10^, ^11^ Trends were defined as significantly increasing or decreasing based on a two‐tailed t‐test with a *p*‐value threshold of <.05 indicating statistical significance. However, due to the suppression of yearly mortality data caused by low death counts, we could not assess temporal mortality shifts in ethnic and racial subpopulations.

Institutional Review Board approval was not necessary given our analyses utilized anonymized and Government‐issued publicly available data from the CDC repository.

## 3 RESULTS

**FIGURE 1:**
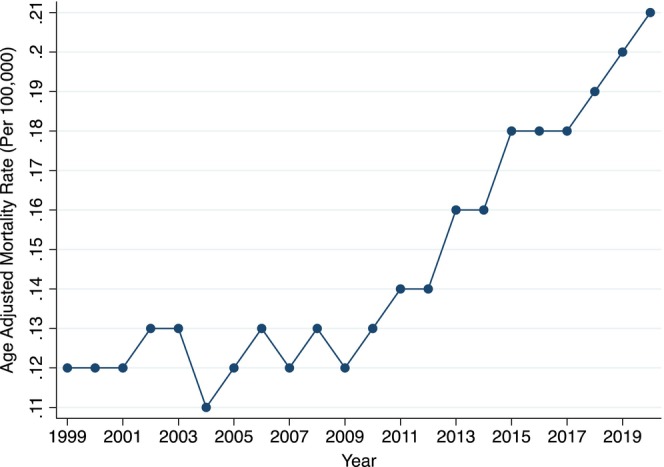
Annual mortality. Figure depicts annual cumulative GIB AAMR in decedents with known atrial fibrillation. AAMR, age‐adjusted mortality rate per 100,000 population; GIB, gastrointestinal bleed.

**FIGURE 2:**
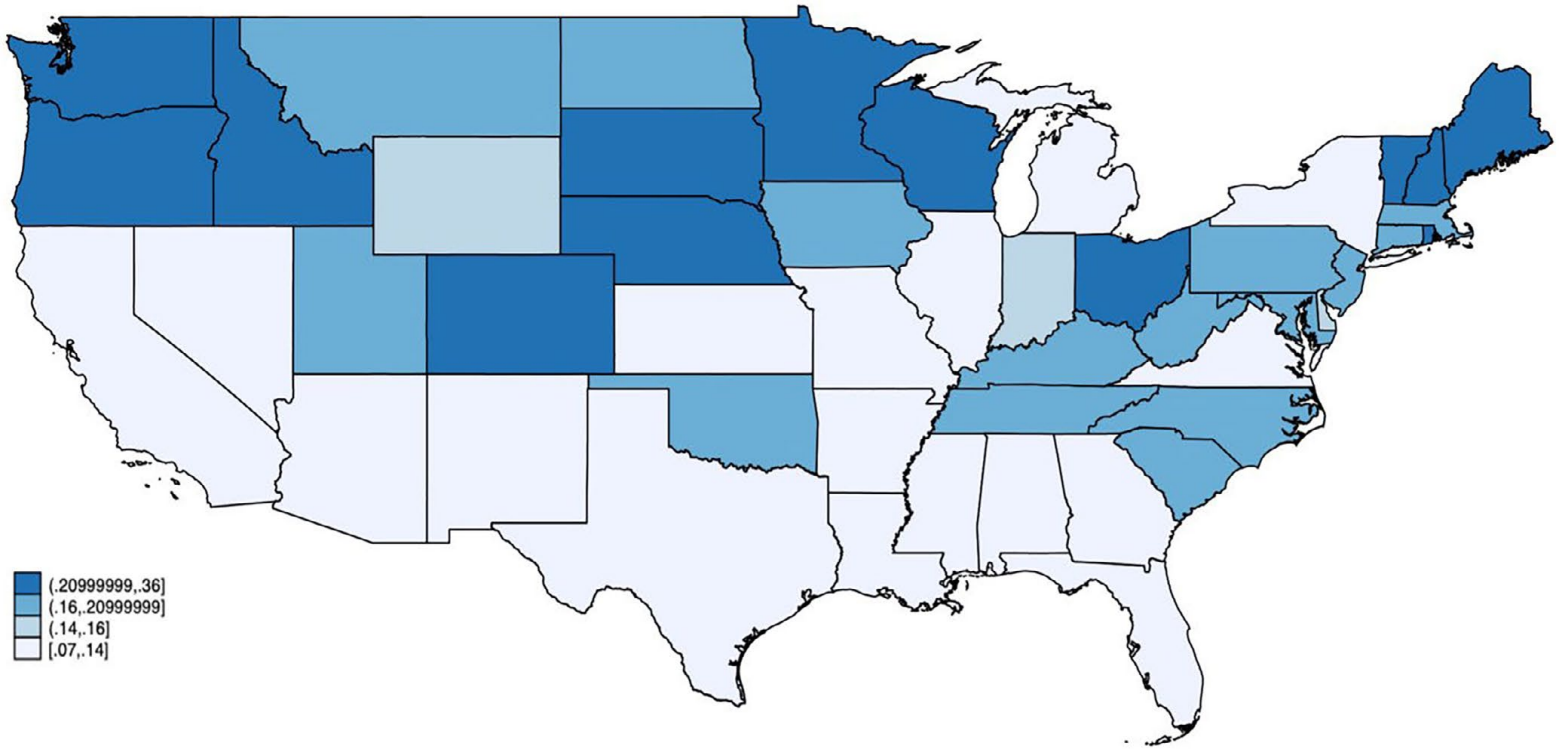
US chloropleth map. Figure depicts cumulative GIB AAMR in decedents with known atrial fibrillation, stratified by states. AAMR, age‐adjusted mortality rate per 100,000 population; GIB, gastrointestinal bleed; US, United States.

**FIGURE 3:**
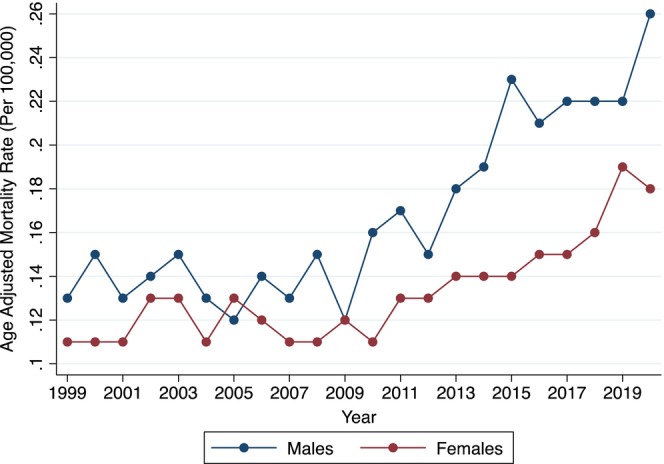
Annual mortality by sex. Figure depicts annual GIB AAMR in decedents with known atrial fibrillation, stratified by sex. AAMR, age‐adjusted mortality rate per 100,000 population; GIB, gastrointestinal bleed.

From 1999 to 2020, there were a total of 11,209 GIB deaths in individuals with underlying AF (Table S2). The AAMR increased from 0.12 (95% CI, 0.10–0.13) in 1999 to 0.21 (95% CI, 0.20–0.23) in 2020 (Figure 1). GIB mortality remained stagnant from 1999 to 2009 (APC +0.5, *p* = .397); whereas it increased from 2009 to 2020 (APC +4.8, *p* < .001). The cumulative AAMR over this 22‐year time period was 0.14 (95% CI, 0.14–0.15) (Figure 2). Mortality was higher in males (AAMR 0.18 [95% CI, 0.18–0.19]) compared to females (0.13 [95% CI, 0.13–0.14]). Among male populations, mortality remained stagnant from 1999 to 2007 (APC –0.49, *p* = .771) followed by an increase from 2007 to 2020 (APC +5.0, *p* < .001). Similarly, mortality among female populations remained stagnant from 1999 to 2010 (APC +0.02, *p* = .983) followed by an increase from 2010 to 2020 (APC +4.45, *p* < .001) (Figure 3).

Non‐Hispanic populations (0.15 [95% CI, 0.15–0.15]) were disproportionately impacted compared to Hispanic populations (AAMR 0.07 [95% CI, 0.06–0.07]). Among the non‐Hispanic populations, White populations (AAMR 0.15 [95% CI, 0.15–0.16]) had the highest mortality rates, followed by Black (AAMR 0.10 [95% CI, 0.09–0.11]), Asian/Pacific Islander (AAMR 0.08 [95% CI, 0.07–0.09]), and American Indian/Alaska Native (AAMR 0.06 [95% CI, 0.04–0.10]) populations.

**FIGURE 4:**
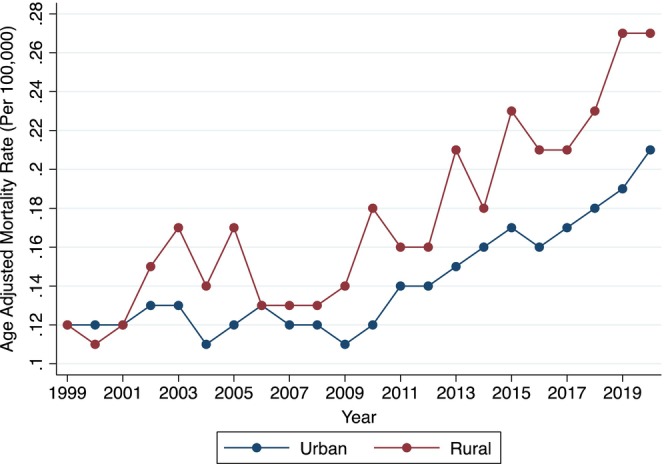
Annual mortality by urbanization. Figure depicts annual GIB AAMR in decedents with known atrial fibrillation, stratified by urbanization. AAMR, age‐adjusted mortality rate per 100,000 population; GIB, gastrointestinal bleed.

Mortality rates were higher in rural regions (AAMR 0.18 [95% CI, 0.18–0.19]) compared to urban regions (AAMR 0.14 [95% CI, 0.14–0.14]). Mortality shifts in urban regions remained stagnant from 1999 to 2009 (APC –0.15, *p* = .806) followed by an increase from 2009 to 2020 (APC +4.83, *p* < .001). However, mortality increased consistently from 1999 to 2020 in rural regions (APC +4.08, *p* < .001) (Figure 4). Among the US census regions, the Northeast (AAMR 0.18 [95% CI, 0.17–0.18]) had the highest mortality rates, followed by the Midwest (AAMR 0.16 [95% CI, 0.15–0.17]), West (AAMR 0.14 [95% CI, 0.13–0.14]), and South (AAMR 0.13 [95% CI, 0.13–0.14]). Mortality among the Northeast remained stagnant from 1999 to 2013 (APC +0.17, *p* = .759) followed by an increase from 2013 to 2020 (APC +5.39, *p* = .001). Within Midwestern regions, mortality increased from 1999 to 2011 (APC +1.28, *p* = .047) followed by a prominent accelerating mortality shift (APC 6.50, *p* < .001). In Southern regions, mortality remained stagnant from 1999 to 2009 (APC +0.73, *p* = .517), followed by an increase from 2009 to 2020 (APC +5.38, *p* < .001). Mortality increased consistently from 1999 to 2020 within Western regions (APC +2.87, *p* < .001).

## 4 DISCUSSION

We aimed to investigate the mortality trends and associated disparities related to GIB in patients with underlying AF. Findings from our study indicate that the annual GIB‐related AAMR in decedents with AF increased between 1999 and 2020. Between 1999 and 2009, the APC in GIB mortality remained relatively stable but significantly increased between 2009 and 2020. GIB mortality in decedents with AF was higher among non‐Hispanic populations, particularly among White and Black subpopulations. Furthermore, rural and Northeastern regions were impacted by the highest GIB mortality rates. These findings provide valuable epidemiological insight into GIB mortality disparities in decedents with AF in the US.

From 1999 to 2020, we observed a dichotomy in mortality trends related to GIB, with distinct periods before and after 2009. Initially, the APC in mortality rates held steady from 1999 to 2009; however, starting in 2009, we noted a significant upward shift in the APC. The increase in GIB mortality during this period may be related to an increase in anticoagulant therapy, particularly within the first 12 months of new AF diagnosis, and improved adherence.^12^ The use of DOACs increased significantly between 2011 and 2020, likely contributing to the increase in adverse outcomes including GIB.^13^, ^14^, ^15^, ^16^, ^17^, ^18^ Therefore, this increased adoption of oral anticoagulation in the latter half of our study period could potentially account for the rising GIB mortality that our analysis has revealed; however, these analyses did not differentiate the type of anticoagulation (i.e., warfarin versus DOACs).

Our results revealed a higher mortality rate from GIB in male patients with AF compared to females, a finding that is consistent with the results of several other studies.^13^, ^19^, ^20^, ^21^, ^22^, ^23^ This disparity is likely due to differences between sex‐specific vulnerability in GIB outcomes, the prevalence of anticoagulant use, and the adverse outcomes encountered.^24^, ^25^, ^26^, ^27^, ^28^, ^29^ For example, male populations have been found to have higher mortality rates in the setting of a GIB compared to female populations.^24^, ^25^ While female populations have a lower likelihood of major bleeding events and overall mortality when using DOACs compared to male populations, they are also less frequently started on anticoagulant therapy.^26^, ^27^, ^28^, ^29^ Furthermore, our analysis revealed that non‐Hispanic individuals, especially those who are non‐Hispanic White, exhibited the highest AAMRs due to GIB when compared to other racial groups. This aligns with data showing that White populations are more likely to be initiated on anticoagulant therapy.^30^, ^31^, ^32^ Moreover, racial differences in how populations respond to anticoagulants may influence these outcomes. For instance, Asian/Pacific Islander groups have been found to have a higher incidence of intracerebral hemorrhage in association with AF treatment.^33^ These findings suggest that race‐specific responses to anticoagulant therapy may significantly impact GIB mortality rates.

Rural areas exhibited higher GIB mortality rates among patients with known AF compared to their urban counterparts. This discrepancy is influenced by various factors, such as the availability of specialized healthcare providers like gastroenterologists and general surgeons, as well as the overall accessibility to healthcare services in rural regions of the US.^34^ Procedures that are crucial in the management of GIB, such as colonoscopies, endoscopies, and endoscopic hemostasis techniques, are more commonly performed in urban hospitals compared to rural hospitals.^35^, ^36^ Additionally, there is a regional variation in anticoagulant therapy prescriptions, with the Northeastern and Midwestern areas reporting higher usage rates.^37^, ^38^, ^39^ This correlates with our findings of increased GIB mortality in these regions, suggesting a link between anticoagulant use and mortality outcomes.

The findings of our study highlights the critical issue of GIB mortality among patients with AF. Oral anticoagulants, while central to AF management, present a significant risk for severe bleeding events, with GIB being the most common site for hemorrhages related to these medications.^40^, ^41^, ^42^ This concern is particularly relevant given the aging US population and the increasing incidence of AF, which together suggest a likely rise in anticoagulant‐related GIBs.^43^, ^44^, ^45^ Recognizing the importance of anticoagulation in treating AF, our study provides epidemiological insight regarding the associated risks with anticoagulation in AF, allowing for greater opportunities to develop targeted preventive strategies to mitigate these disparities and to urge policy‐makers and healthcare personnel epidemiologists to evaluate for potential etiologies and data‐driven evidence‐based policy updates. These GIB mortality disparities also emphasize the need for personalized treatment approaches and risk assessments in individuals with AF. Through the use of leveraging advancements in drug development and clinical data, patient education, and monitoring protocols, these disparities may potentially be mitigated in vulnerable populations.

This manuscript has limitations that should be considered. The analysis did not distinguish between different types and locations of GIB, which limits our ability to conduct a nuanced analysis of specific GIB types and the disparities in associated mortality. Additionally, the use of death certificates to classify decedents, with GIB as the underlying cause and AF as a contributing cause, does not account for individual‐level data such as anticoagulant use. Therefore, it is possible that not all included decedents were receiving anticoagulation therapy. Given that CDC repository offers data at an aggregate level, receiving patient‐level data would not be feasible, which also precludes the ability to conduct a multivariate analysis. Another limitation is regarding the validation of the mortality data reported in the CDC database. To the best of our knowledge, there has been no study to date that has validated the CDC repository‐reported causes of death within the realm of cardiovascular disease. One study in 2014 demonstrated that the population‐level mortality data within the CDC consistently aligned with clinical information found in medical records.^46^ Another potential issue is the risk of misclassification errors inherent in the use of death certificate data. Despite these limitations, the strength of our analysis is related to the utilization of the CDC comprehensive repository, a database that is known to account for over 99% of all deaths in the US, allowing representativeness and national relevance to our findings.^47^

## 5 CONCLUSIONS

GIB mortality disparities among individuals with AF exist in the United States, particularly among males, non‐Hispanic White populations, and within rural and Northeastern US regions. These variations in mortality emphasize the necessity for targeted healthcare interventions. Through the identification of these vulnerable populations, efforts can be focused on achieving more equitable healthcare outcomes.

## FUNDING INFORMATION

This research did not receive any specific grant from funding agencies in the public, commercial, or not‐for‐profit sectors.

## CONFLICT OF INTEREST STATEMENT

Authors have no conflict of interest.

## ETHICS STATEMENT

Ethical approval is not required given the use of government‐issued anonymized data.

## Supporting information


**Table S1.** GIB ICD‐10 codes. All ICD‐10 codes related to gastrointestinal bleeds that were queried as the underlying cause of death in decedents with atrial fibrillation.
**Table S2.** Yearly mortality data. Annual death counts, population size, and crude‐ and age‐adjusted mortality rates related to GIB deaths in decedents with atrial fibrillation.

## Data Availability

All data are available in publicly available repositories.
