# Supplementary material for: Gastrointestinal bleed mortality disparities in patients with atrial fibrillation: A cross‐sectional analysis 1999–2020
Source: J Arrhythm. 2025 Jan 14;41(1):e13223. doi: 10.1002/joa3.13223 (PMC11730723; doi:10.1002/joa3.13223)
Supplement: Supplementary file 1 — Table S1. GIB ICD‐10 codes. All ICD‐10 codes related to gastrointestinal bleeds that were queried as the underlying cause of death in decedents with atrial fibrillation. Table S2. Yearly mortality data. Annual death counts, population size, and crude‐ and age‐adjusted mortality rates related to GIB deaths in decedents with atrial fibrillation. [file JOA3-41-e13223-s001.docx]

**Table S1. GIB ICD-10 Codes.** All ICD-10 codes related to gastrointestinal bleeds that were queried as the underlying cause of death in decedents with atrial fibrillation.

| **GI Bleed ICD-10 Codes** |
| --- |
| K25.0 |
| K25.2 |
| K25.4 |
| K25.6 |
| K26.0 |
| K26.2 |
| K26.4 |
| K26.6 |
| K27.0 |
| K27.2 |
| K27.4 |
| K27.6 |
| K28.0 |
| K28.2 |
| K28.4 |
| K28.6 |
| K28.9 |
| K29.0 |
| K62.5 |
| K92.0 |
| K92.1 |
| K92.2 |

Abbreviations: GIB=gastrointestinal bleed, ICD-10=International Classification of Diseases, Tenth Revision.

**Table S2. Yearly Mortality Data.** Annual death counts, population size, and crude- and age-adjusted mortality rates related to GIB deaths in decedents with atrial fibrillation.

| **Year** | **Deaths** | | **Population** | **CMR (95% CI)** | **AAMR (95% CI)** |
| --- | --- | --- | --- | --- | --- |
| **Cumulative** | | | | | |
| 1999 | 327 | 279,040,168 | | 0.12 (0.1-0.13) | 0.12 (0.1-0.13) |
| 2000 | 338 | 281,421,906 | | 0.12 (0.11-0.13) | 0.12 (0.11-0.13) |
| 2001 | 338 | 284,968,955 | | 0.12 (0.11-0.13) | 0.12 (0.1-0.13) |
| 2002 | 390 | 287,625,193 | | 0.14 (0.12-0.15) | 0.13 (0.12-0.15) |
| 2003 | 399 | 290,107,933 | | 0.14 (0.12-0.15) | 0.13 (0.12-0.15) |
| 2004 | 344 | 292,805,298 | | 0.12 (0.11-0.13) | 0.11 (0.1-0.13) |
| 2005 | 386 | 295,516,599 | | 0.13 (0.12-0.14) | 0.12 (0.11-0.14) |
| 2006 | 405 | 298,379,912 | | 0.14 (0.12-0.15) | 0.13 (0.12-0.15) |
| 2007 | 385 | 301,231,207 | | 0.13 (0.12-0.14) | 0.12 (0.11-0.14) |
| 2008 | 410 | 304,093,966 | | 0.13 (0.12-0.15) | 0.13 (0.12-0.14) |
| 2009 | 404 | 306,771,529 | | 0.13 (0.12-0.14) | 0.12 (0.11-0.13) |
| 2010 | 435 | 308,745,538 | | 0.14 (0.13-0.15) | 0.13 (0.12-0.14) |
| 2011 | 487 | 311,591,917 | | 0.16 (0.14-0.17) | 0.14 (0.13-0.15) |
| 2012 | 494 | 313,914,040 | | 0.16 (0.14-0.17) | 0.14 (0.13-0.15) |
| 2013 | 575 | 316,128,839 | | 0.18 (0.17-0.2) | 0.16 (0.14-0.17) |
| 2014 | 586 | 318,857,056 | | 0.18 (0.17-0.2) | 0.16 (0.15-0.18) |
| 2015 | 670 | 321,418,820 | | 0.21 (0.19-0.22) | 0.18 (0.17-0.2) |
| 2016 | 666 | 323,127,513 | | 0.21 (0.19-0.22) | 0.18 (0.16-0.19) |
| 2017 | 699 | 325,719,178 | | 0.21 (0.2-0.23) | 0.18 (0.17-0.19) |
| 2018 | 757 | 327,167,434 | | 0.23 (0.21-0.25) | 0.19 (0.17-0.2) |
| 2019 | 816 | 328,239,523 | | 0.25 (0.23-0.27) | 0.2 (0.18-0.21) |
| 2020 | 898 | 329,484,123 | | 0.27 (0.25-0.29) | 0.21 (0.2-0.23) |
| **Males** | | | | | |
| 1999 | 126 | 136,802,873 | | 0.09 (0.08-0.11) | 0.13 (0.11-0.15) |
| 2000 | 141 | 138,053,563 | | 0.1 (0.09-0.12) | 0.15 (0.12-0.17) |
| 2001 | 131 | 139,891,492 | | 0.09 (0.08-0.11) | 0.13 (0.11-0.16) |
| 2002 | 143 | 141,230,559 | | 0.1 (0.08-0.12) | 0.14 (0.12-0.17) |
| 2003 | 156 | 142,428,897 | | 0.11 (0.09-0.13) | 0.15 (0.12-0.17) |
| 2004 | 147 | 143,828,012 | | 0.1 (0.09-0.12) | 0.13 (0.11-0.16) |
| 2005 | 136 | 145,197,078 | | 0.09 (0.08-0.11) | 0.12 (0.1-0.14) |
| 2006 | 168 | 146,647,265 | | 0.11 (0.1-0.13) | 0.14 (0.12-0.17) |
| 2007 | 152 | 148,064,854 | | 0.1 (0.09-0.12) | 0.13 (0.11-0.15) |
| 2008 | 181 | 149,489,951 | | 0.12 (0.1-0.14) | 0.15 (0.13-0.17) |
| 2009 | 149 | 150,807,454 | | 0.1 (0.08-0.11) | 0.12 (0.1-0.14) |
| 2010 | 193 | 151,781,326 | | 0.13 (0.11-0.15) | 0.16 (0.14-0.18) |
| 2011 | 222 | 153,290,819 | | 0.14 (0.13-0.16) | 0.17 (0.15-0.19) |
| 2012 | 202 | 154,492,067 | | 0.13 (0.11-0.15) | 0.15 (0.13-0.17) |
| 2013 | 258 | 155,651,602 | | 0.17 (0.15-0.19) | 0.18 (0.15-0.2) |
| 2014 | 272 | 156,936,487 | | 0.17 (0.15-0.19) | 0.19 (0.17-0.21) |
| 2015 | 340 | 158,229,297 | | 0.21 (0.19-0.24) | 0.23 (0.21-0.26) |
| 2016 | 304 | 159,078,923 | | 0.19 (0.17-0.21) | 0.21 (0.18-0.23) |
| 2017 | 345 | 160,408,119 | | 0.22 (0.19-0.24) | 0.22 (0.2-0.24) |
| 2018 | 368 | 161,128,679 | | 0.23 (0.21-0.25) | 0.22 (0.2-0.25) |
| 2019 | 375 | 161,657,324 | | 0.23 (0.21-0.26) | 0.22 (0.2-0.25) |
| 2020 | 436 | 162,256,202 | | 0.27 (0.24-0.29) | 0.26 (0.23-0.28) |
| **Females** | | | | | |
| 1999 | 201 | 142,237,295 | | 0.14 (0.12-0.16) | 0.11 (0.09-0.12) |
| 2000 | 197 | 143,368,343 | | 0.14 (0.12-0.16) | 0.11 (0.09-0.12) |
| 2001 | 207 | 145,077,463 | | 0.14 (0.12-0.16) | 0.11 (0.09-0.12) |
| 2002 | 247 | 146,394,634 | | 0.17 (0.15-0.19) | 0.13 (0.11-0.15) |
| 2003 | 243 | 147,679,036 | | 0.16 (0.14-0.19) | 0.13 (0.11-0.15) |
| 2004 | 197 | 148,977,286 | | 0.13 (0.11-0.15) | 0.11 (0.09-0.12) |
| 2005 | 250 | 150,319,521 | | 0.17 (0.15-0.19) | 0.13 (0.11-0.14) |
| 2006 | 237 | 151,732,647 | | 0.16 (0.14-0.18) | 0.12 (0.11-0.14) |
| 2007 | 233 | 153,166,353 | | 0.15 (0.13-0.17) | 0.11 (0.09-0.12) |
| 2008 | 229 | 154,604,015 | | 0.15 (0.13-0.17) | 0.11 (0.1-0.13) |
| 2009 | 255 | 155,964,075 | | 0.16 (0.14-0.18) | 0.12 (0.11-0.14) |
| 2010 | 242 | 156,964,212 | | 0.15 (0.13-0.17) | 0.11 (0.1-0.13) |
| 2011 | 265 | 158,301,098 | | 0.17 (0.15-0.19) | 0.13 (0.11-0.14) |
| 2012 | 292 | 159,421,973 | | 0.18 (0.16-0.2) | 0.13 (0.11-0.14) |
| 2013 | 317 | 160,477,237 | | 0.2 (0.18-0.22) | 0.14 (0.12-0.15) |
| 2014 | 314 | 161,920,569 | | 0.19 (0.17-0.22) | 0.14 (0.13-0.16) |
| 2015 | 330 | 163,189,523 | | 0.2 (0.18-0.22) | 0.14 (0.12-0.15) |
| 2016 | 362 | 164,048,590 | | 0.22 (0.2-0.24) | 0.15 (0.13-0.17) |
| 2017 | 354 | 165,311,059 | | 0.21 (0.19-0.24) | 0.15 (0.14-0.17) |
| 2018 | 389 | 166,038,755 | | 0.23 (0.21-0.26) | 0.16 (0.14-0.17) |
| 2019 | 441 | 166,582,199 | | 0.26 (0.24-0.29) | 0.19 (0.17-0.2) |
| 2020 | 462 | 167,227,921 | | 0.28 (0.25-0.3) | 0.18 (0.17-0.2) |
| **Urban Regions** | | | | | |
| 1999 | 261 | 234,421,194 | | 0.11 (0.1-0.12) | 0.12 (0.11-0.14) |
| 2000 | 278 | 236,637,796 | | 0.12 (0.1-0.13) | 0.12 (0.11-0.14) |
| 2001 | 273 | 240,128,266 | | 0.11 (0.1-0.13) | 0.12 (0.1-0.13) |
| 2002 | 308 | 242,665,792 | | 0.13 (0.11-0.14) | 0.13 (0.12-0.15) |
| 2003 | 305 | 244,993,933 | | 0.12 (0.11-0.14) | 0.13 (0.12-0.15) |
| 2004 | 262 | 247,510,633 | | 0.11 (0.09-0.12) | 0.11 (0.09-0.12) |
| 2005 | 292 | 250,039,935 | | 0.12 (0.1-0.13) | 0.12 (0.1-0.13) |
| 2006 | 328 | 252,641,624 | | 0.13 (0.12-0.14) | 0.13 (0.11-0.14) |
| 2007 | 311 | 255,300,617 | | 0.12 (0.11-0.14) | 0.12 (0.1-0.13) |
| 2008 | 333 | 257,996,520 | | 0.13 (0.12-0.14) | 0.12 (0.11-0.14) |
| 2009 | 320 | 260,573,371 | | 0.12 (0.11-0.14) | 0.11 (0.1-0.13) |
| 2010 | 330 | 262,452,132 | | 0.13 (0.11-0.14) | 0.12 (0.1-0.13) |
| 2011 | 392 | 265,270,604 | | 0.15 (0.13-0.16) | 0.14 (0.12-0.15) |
| 2012 | 400 | 267,664,440 | | 0.15 (0.13-0.16) | 0.14 (0.12-0.15) |
| 2013 | 435 | 269,911,242 | | 0.16 (0.15-0.18) | 0.15 (0.14-0.16) |
| 2014 | 465 | 272,667,942 | | 0.17 (0.16-0.19) | 0.16 (0.14-0.17) |
| 2015 | 528 | 275,252,217 | | 0.19 (0.18-0.21) | 0.17 (0.16-0.19) |
| 2016 | 530 | 277,016,929 | | 0.19 (0.18-0.21) | 0.16 (0.15-0.18) |
| 2017 | 561 | 279,636,439 | | 0.2 (0.18-0.22) | 0.17 (0.16-0.19) |
| 2018 | 603 | 281,067,210 | | 0.21 (0.2-0.23) | 0.18 (0.17-0.19) |
| 2019 | 632 | 282,176,462 | | 0.22 (0.21-0.24) | 0.19 (0.17-0.2) |
| 2020 | 718 | 283,450,351 | | 0.25 (0.23-0.27) | 0.21 (0.19-0.22) |
| **Rural Regions** | | | | | |
| 1999 | 66 | 44,618,974 | | 0.15 (0.11-0.19) | 0.12 (0.09-0.15) |
| 2000 | 60 | 44,784,110 | | 0.13 (0.1-0.17) | 0.11 (0.08-0.14) |
| 2001 | 65 | 44,840,689 | | 0.14 (0.11-0.18) | 0.12 (0.09-0.15) |
| 2002 | 82 | 44,959,401 | | 0.18 (0.15-0.23) | 0.15 (0.12-0.19) |
| 2003 | 94 | 45,114,000 | | 0.21 (0.17-0.25) | 0.17 (0.14-0.21) |
| 2004 | 82 | 45,294,665 | | 0.18 (0.14-0.22) | 0.14 (0.11-0.18) |
| 2005 | 94 | 45,476,664 | | 0.21 (0.17-0.25) | 0.17 (0.13-0.2) |
| 2006 | 77 | 45,738,288 | | 0.17 (0.13-0.21) | 0.13 (0.1-0.17) |
| 2007 | 74 | 45,930,590 | | 0.16 (0.13-0.2) | 0.13 (0.1-0.16) |
| 2008 | 77 | 46,097,446 | | 0.17 (0.13-0.21) | 0.13 (0.1-0.16) |
| 2009 | 84 | 46,198,158 | | 0.18 (0.15-0.23) | 0.14 (0.12-0.18) |
| 2010 | 105 | 46,293,406 | | 0.23 (0.18-0.27) | 0.18 (0.15-0.22) |
| 2011 | 95 | 46,321,313 | | 0.21 (0.17-0.25) | 0.16 (0.13-0.19) |
| 2012 | 94 | 46,249,600 | | 0.2 (0.16-0.25) | 0.16 (0.13-0.2) |
| 2013 | 140 | 46,217,597 | | 0.3 (0.25-0.35) | 0.21 (0.18-0.25) |
| 2014 | 121 | 46,189,114 | | 0.26 (0.22-0.31) | 0.18 (0.15-0.22) |
| 2015 | 142 | 46,166,603 | | 0.31 (0.26-0.36) | 0.23 (0.19-0.26) |
| 2016 | 136 | 46,110,584 | | 0.29 (0.25-0.34) | 0.21 (0.18-0.25) |
| 2017 | 138 | 46,082,739 | | 0.3 (0.25-0.35) | 0.21 (0.17-0.24) |
| 2018 | 154 | 46,100,224 | | 0.33 (0.28-0.39) | 0.23 (0.19-0.27) |
| 2019 | 184 | 46,063,061 | | 0.4 (0.34-0.46) | 0.27 (0.23-0.31) |
| 2020 | 180 | 46,024,426 | | 0.39 (0.33-0.45) | 0.27 (0.23-0.32) |
| **Northeast** | | | | | |
| 1999 | 93 | 53,343,775 | | 0.17 (0.14-0.21) | 0.16 (0.13-0.19) |
| 2000 | 87 | 53,594,378 | | 0.16 (0.13-0.2) | 0.15 (0.12-0.18) |
| 2001 | 99 | 53,915,522 | | 0.18 (0.15-0.22) | 0.16 (0.13-0.19) |
| 2002 | 105 | 54,143,915 | | 0.19 (0.16-0.23) | 0.17 (0.14-0.21) |
| 2003 | 110 | 54,334,453 | | 0.2 (0.16-0.24) | 0.18 (0.14-0.21) |
| 2004 | 99 | 54,423,533 | | 0.18 (0.15-0.22) | 0.16 (0.13-0.19) |
| 2005 | 99 | 54,451,230 | | 0.18 (0.15-0.22) | 0.15 (0.12-0.18) |
| 2006 | 107 | 54,522,659 | | 0.2 (0.16-0.23) | 0.16 (0.13-0.2) |
| 2007 | 100 | 54,653,362 | | 0.18 (0.15-0.22) | 0.15 (0.12-0.18) |
| 2008 | 96 | 54,875,926 | | 0.17 (0.14-0.21) | 0.15 (0.12-0.18) |
| 2009 | 104 | 55,133,101 | | 0.19 (0.15-0.22) | 0.15 (0.12-0.18) |
| 2010 | 104 | 55,317,240 | | 0.19 (0.15-0.22) | 0.15 (0.12-0.18) |
| 2011 | 133 | 55,521,598 | | 0.24 (0.2-0.28) | 0.19 (0.15-0.22) |
| 2012 | 131 | 55,761,091 | | 0.23 (0.19-0.28) | 0.18 (0.14-0.21) |
| 2013 | 125 | 55,943,073 | | 0.22 (0.18-0.26) | 0.16 (0.13-0.19) |
| 2014 | 132 | 56,152,333 | | 0.24 (0.19-0.28) | 0.18 (0.15-0.21) |
| 2015 | 143 | 56,283,891 | | 0.25 (0.21-0.3) | 0.19 (0.16-0.22) |
| 2016 | 136 | 56,209,510 | | 0.24 (0.2-0.28) | 0.17 (0.14-0.2) |
| 2017 | 148 | 56,470,581 | | 0.26 (0.22-0.3) | 0.2 (0.17-0.23) |
| 2018 | 168 | 56,111,079 | | 0.3 (0.25-0.34) | 0.21 (0.18-0.24) |
| 2019 | 174 | 55,982,803 | | 0.31 (0.26-0.36) | 0.22 (0.19-0.25) |
| 2020 | 194 | 55,849,869 | | 0.35 (0.3-0.4) | 0.24 (0.2-0.27) |
| **Midwest** | | | | | |
| 1999 | 76 | 64,100,061 | | 0.12 (0.09-0.15) | 0.11 (0.09-0.14) |
| 2000 | 92 | 64,392,776 | | 0.14 (0.12-0.18) | 0.13 (0.11-0.16) |
| 2001 | 85 | 64,776,531 | | 0.13 (0.1-0.16) | 0.13 (0.1-0.16) |
| 2002 | 102 | 65,018,293 | | 0.16 (0.13-0.19) | 0.14 (0.12-0.17) |
| 2003 | 96 | 65,276,954 | | 0.15 (0.12-0.18) | 0.13 (0.11-0.16) |
| 2004 | 95 | 65,532,305 | | 0.14 (0.12-0.18) | 0.14 (0.11-0.17) |
| 2005 | 95 | 65,751,872 | | 0.14 (0.12-0.18) | 0.13 (0.11-0.16) |
| 2006 | 108 | 66,028,555 | | 0.16 (0.13-0.19) | 0.14 (0.12-0.17) |
| 2007 | 105 | 66,293,689 | | 0.16 (0.13-0.19) | 0.13 (0.11-0.16) |
| 2008 | 112 | 66,523,935 | | 0.17 (0.14-0.2) | 0.14 (0.12-0.17) |
| 2009 | 111 | 66,748,437 | | 0.17 (0.14-0.2) | 0.14 (0.11-0.17) |
| 2010 | 122 | 66,927,001 | | 0.18 (0.15-0.21) | 0.15 (0.13-0.18) |
| 2011 | 119 | 67,158,835 | | 0.18 (0.15-0.21) | 0.14 (0.12-0.17) |
| 2012 | 111 | 67,316,297 | | 0.16 (0.13-0.2) | 0.13 (0.11-0.16) |
| 2013 | 151 | 67,547,890 | | 0.22 (0.19-0.26) | 0.18 (0.15-0.21) |
| 2014 | 152 | 67,745,108 | | 0.22 (0.19-0.26) | 0.18 (0.15-0.21) |
| 2015 | 173 | 67,907,403 | | 0.25 (0.22-0.29) | 0.21 (0.17-0.24) |
| 2016 | 168 | 67,941,429 | | 0.25 (0.21-0.28) | 0.2 (0.17-0.23) |
| 2017 | 175 | 68,179,351 | | 0.26 (0.22-0.29) | 0.2 (0.17-0.23) |
| 2018 | 202 | 68,308,744 | | 0.3 (0.25-0.34) | 0.22 (0.19-0.25) |
| 2019 | 206 | 68,329,004 | | 0.3 (0.26-0.34) | 0.23 (0.2-0.26) |
| 2020 | 231 | 68,316,744 | | 0.34 (0.29-0.38) | 0.26 (0.22-0.29) |
| **South** | | | | | |
| 1999 | 100 | 99,164,460 | | 0.1 (0.08-0.12) | 0.1 (0.08-0.12) |
| 2000 | 101 | 100,236,820 | | 0.1 (0.08-0.12) | 0.1 (0.08-0.12) |
| 2001 | 99 | 101,849,575 | | 0.1 (0.08-0.12) | 0.1 (0.08-0.12) |
| 2002 | 106 | 103,150,787 | | 0.1 (0.08-0.12) | 0.1 (0.08-0.12) |
| 2003 | 124 | 104,380,188 | | 0.12 (0.1-0.14) | 0.12 (0.1-0.15) |
| 2004 | 92 | 105,883,977 | | 0.09 (0.07-0.11) | 0.09 (0.07-0.11) |
| 2005 | 114 | 107,479,771 | | 0.11 (0.09-0.13) | 0.11 (0.09-0.13) |
| 2006 | 109 | 109,076,933 | | 0.1 (0.08-0.12) | 0.1 (0.08-0.12) |
| 2007 | 109 | 110,688,742 | | 0.1 (0.08-0.12) | 0.1 (0.08-0.12) |
| 2008 | 122 | 112,184,930 | | 0.11 (0.09-0.13) | 0.11 (0.09-0.13) |
| 2009 | 121 | 113,548,615 | | 0.11 (0.09-0.13) | 0.11 (0.09-0.13) |
| 2010 | 118 | 114,555,744 | | 0.1 (0.08-0.12) | 0.09 (0.08-0.11) |
| 2011 | 147 | 116,046,736 | | 0.13 (0.11-0.15) | 0.12 (0.1-0.14) |
| 2012 | 160 | 117,257,221 | | 0.14 (0.12-0.16) | 0.13 (0.11-0.15) |
| 2013 | 189 | 118,383,453 | | 0.16 (0.14-0.18) | 0.14 (0.12-0.17) |
| 2014 | 176 | 119,771,934 | | 0.15 (0.13-0.17) | 0.13 (0.11-0.15) |
| 2015 | 213 | 121,182,847 | | 0.18 (0.15-0.2) | 0.16 (0.14-0.18) |
| 2016 | 222 | 122,319,574 | | 0.18 (0.16-0.21) | 0.17 (0.15-0.19) |
| 2017 | 246 | 123,658,624 | | 0.2 (0.17-0.22) | 0.17 (0.15-0.19) |
| 2018 | 238 | 124,753,948 | | 0.19 (0.17-0.22) | 0.17 (0.14-0.19) |
| 2019 | 266 | 125,580,448 | | 0.21 (0.19-0.24) | 0.17 (0.15-0.19) |
| 2020 | 286 | 126,662,754 | | 0.23 (0.2-0.25) | 0.19 (0.17-0.21) |
| **West** | | | | | |
| 1999 | 58 | 62,431,872 | | 0.09 (0.07-0.12) | 0.11 (0.08-0.14) |
| 2000 | 58 | 63,197,932 | | 0.09 (0.07-0.12) | 0.11 (0.08-0.14) |
| 2001 | 55 | 64,427,327 | | 0.09 (0.06-0.11) | 0.1 (0.08-0.14) |
| 2002 | 77 | 65,312,198 | | 0.12 (0.09-0.15) | 0.14 (0.11-0.17) |
| 2003 | 69 | 66,116,338 | | 0.1 (0.08-0.13) | 0.12 (0.09-0.15) |
| 2004 | 58 | 66,965,483 | | 0.09 (0.07-0.11) | 0.1 (0.07-0.13) |
| 2005 | 78 | 67,833,726 | | 0.11 (0.09-0.14) | 0.12 (0.1-0.16) |
| 2006 | 81 | 68,751,765 | | 0.12 (0.09-0.15) | 0.13 (0.1-0.16) |
| 2007 | 71 | 69,595,414 | | 0.1 (0.08-0.13) | 0.11 (0.08-0.14) |
| 2008 | 80 | 70,509,175 | | 0.11 (0.09-0.14) | 0.12 (0.09-0.15) |
| 2009 | 68 | 71,341,376 | | 0.1 (0.07-0.12) | 0.1 (0.07-0.12) |
| 2010 | 91 | 71,945,553 | | 0.13 (0.1-0.16) | 0.13 (0.1-0.16) |
| 2011 | 88 | 72,864,748 | | 0.12 (0.1-0.15) | 0.12 (0.1-0.15) |
| 2012 | 92 | 73,579,431 | | 0.13 (0.1-0.15) | 0.12 (0.1-0.15) |
| 2013 | 110 | 74,254,423 | | 0.15 (0.12-0.18) | 0.14 (0.12-0.17) |
| 2014 | 126 | 75,187,681 | | 0.17 (0.14-0.2) | 0.15 (0.13-0.18) |
| 2015 | 141 | 76,044,679 | | 0.19 (0.15-0.22) | 0.16 (0.13-0.19) |
| 2016 | 140 | 76,657,000 | | 0.18 (0.15-0.21) | 0.17 (0.14-0.2) |
| 2017 | 130 | 77,410,622 | | 0.17 (0.14-0.2) | 0.15 (0.13-0.18) |
| 2018 | 149 | 77,993,663 | | 0.19 (0.16-0.22) | 0.16 (0.14-0.19) |
| 2019 | 170 | 78,347,268 | | 0.22 (0.18-0.25) | 0.19 (0.16-0.22) |
| 2020 | 187 | 78,654,756 | | 0.24 (0.2-0.27) | 0.21 (0.18-0.24) |
